# Adsorption Behavior and Mechanism of Rhodamine B on a Polyvinyl Alcohol/Carboxymethyl Chitosan Hydrogel: Integrated Experimental and Computational Study

**DOI:** 10.3390/molecules31101619

**Published:** 2026-05-11

**Authors:** Shi Yi, Qingyun Li, Xinrui Zhu, Shuxin Li, Ting Hu, Xinyi Huang, Jiazheng Luo, Hongbo Xiao, Yihui Zhou, Bo Wang, Rongkui Su, Xiping Lei

**Affiliations:** 1 Hunan Automotive Engineering Vocational University, Zhuzhou 412001, China; 2 College of Life Science and Technology, Central South University of Forestry and Technology, Changsha 410004, China; 3 College of Ecology and Environment, Central South University of Forestry and Technology, Changsha 410004, China; 4 College of Chemistry and Chemical Engineering, Central South University of Forestry and Technology, Changsha 410004, China; 5 Research Center of Advanced Materials and Green Energy, Jiangxi Institute of Technology, Nanchang 330098, China

**Keywords:** carboxylated chitosan, polyvinyl alcohol, hydrogel, rhodamine B, adsorption performance, removal mechanism

## Abstract

In this study, a polyvinyl alcohol/carboxymethyl chitosan (PVA/CCTS) hydrogel was synthesized via free radical polymerization and employed for the adsorption of Rhodamine B (RhB) from aqueous solution. The hydrogel was systematically characterized by FTIR, SEM, XPS, and BET analyses, confirming its interconnected porous network and functional group composition. Under optimized conditions (adsorbent dosage = 0.1 g, pH = 6, RhB concentration = 65 mg·L^−1^, and *T* = 298.15 ± 2 K), the maximum adsorption capacity reached 15.88 mg·g^−1^. Kinetic analysis showed that the pseudo-second-order model best described the adsorption behavior under optimal conditions, indicating that the uptake of RhB is governed by multiple interaction mechanisms rather than simple physisorption alone. The equilibrium data were best fitted by the Freundlich isotherm (*R*^2^ = 0.976), indicating surface heterogeneity of the hydrogel. Thermodynamic evaluation revealed an endothermic (Δ*H* = 28.38 ± 4.40 kJ·mol^−1^), with adsorption efficiency improving at elevated temperatures. The hydrogel retained appreciable adsorption capacity after three adsorption–desorption cycles (5.78 mg·g^−1^ at the third cycle). Density functional theory (DFT) calculations identified -COOH and -NH_2_ groups as the primary active sites, and molecular electrostatic potential analysis confirmed that electrostatic interactions between the negatively charged hydrogel surface and cationic RhB drive the initial adsorption. Molecular dynamics (MD) simulations over 100 ns further demonstrated that van der Waals forces constitute the dominant driving force, supplemented by electrostatic interactions and hydrogen bonding, with the hydrogel’s cross-linked network stabilizing adsorbed RhB molecules. The integrated experimental computational approach provides a comprehensive mechanistic understanding of RhB adsorption on PVA/CCTS hydrogel, offering guidance for the rational design of polysaccharide-based adsorbents for dye-contaminated wastewater treatment.

## 1. Introduction

Rhodamine B (RhB) is a synthetic cationic fluorescent red dye belonging to the triphenylmethane family. Due to its excellent stability and resistance to biodegradation, it has been widely used as a colorant in the textile industry. However, RhB itself is a carcinogen and a neurotoxin, posing a serious threat to human health. Symptoms of RhB exposure include respiratory tract infections, skin and gastrointestinal irritation, and damage to vital organs such as liver and thyroid gland [1]. The need for effective removal of RhB from water bodies is therefore imperative.

While there are several established methods for RhB removal, including membrane separation [2], photochemical oxidation [3], Fenton pre-oxidation combined with activated sludge [4], and biological degradation [5], each has its distinct advantages and associated limitations. Membrane separation technology offers precise control over membrane pore size, facilitating efficient and selective separation of RhB. However, the high cost and challenges associated with maintaining membrane performance curtail its broader application. Photochemical oxidation effectively breaks down RhB by generating highly reactive free radicals under light exposure through a photocatalyst, but its efficacy for treating high-concentration wastewater requires further enhancement. The integrated process of Fenton pre-oxidation and activated sludge ensures wastewater meets stringent discharge criteria, though its economic implications and technical intricacies cannot be overlooked. While biodegradation is eco-friendly, the complexities of RhB degradation and fluctuating environmental conditions present significant hurdles for conventional biological treatment techniques. Adsorption stands out as a promising method, characterized by its intuitive design, operational simplicity, cost-effectiveness, and minimal chemical requirements [6]. Nonetheless, the non-biodegradable nature and limited adsorption capacity of traditional adsorbents have prompted investigations into innovative adsorbent materials. These are anticipated to offer superior adsorption efficiency, full biodegradability, biocompatibility, and non-toxicity.

Chitosan (CS) has gained popularity in dye adsorbent research due to its abundance, affordability, non-toxicity, superior biocompatibility, and biodegradability. Its molecular structure is rich in active hydroxyl and amino functional groups, giving it unique adsorption properties [7]. For example, Wan et al. [8] created polyethyleneimine-modified chitosan microspheres with a peak adsorption capacity of 400 mg·g^−1^ for methyl orange. Additionally, Flores-Chaparro et al. [9] formulated a chitosan-macroalgae biocomposite with adsorption capacities of 58.68, 16.64, and 6.13 mg·g^−1^ for benzene, toluene, and naphthalene in water, respectively. Traditional chitosan, characterized by the presence of numerous hydrogen bonds, exhibits limited solubility in water. However, its modified form, carboxymethylated chitosan, displays a notable enhancement in water solubility. Additionally, the latter offers an increased number of adsorption sites, presenting a clear advantage over its traditional counterpart. Despite these improvements, chitosan-based materials continue to possess several limitations, including poor chemical stability, suboptimal mechanical properties, low selectivity for metal ions, and a diminished adsorption capacity due to elevated crystallinity [10]. Conversely, polyvinyl alcohol (PVA), endowed with abundant hydroxyl functional groups (-OH) and exceptional flexibility and adhesion, has found extensive applications as a binder, emulsifier [11], and dispersant [12] in various industries such as textiles, food, pharmaceuticals, construction, wood processing, papermaking, printing, agriculture, steel, and polymer chemistry. Moreover, when blended with chitosan, PVA acts as a plasticizer, significantly augmenting the moldability, mechanical strength, and chemical resistance of chitosan-based adsorbents.

Chitosan-based adsorbents, in their powder form, face significant limitations for widespread use in wastewater treatment due to challenges associated with their effective recovery and separation. Hydrogels, a category of polymers with a three-dimensional cross-linked network structure, are distinguished by their high water-retention capacity. Hydrogels were initially developed and most widely applied in the biomedical field (e.g., drug delivery, tissue engineering, and wound dressings) [13]. In recent years, however, their high water content, tunable porosity, three-dimensional cross-linked network structure, and ease of functionalization have garnered increasing attention in environmental remediation, particularly for the adsorption of dyes and heavy metals [14]. Hydrogels are composed of hydrophilic polymer networks and are predominantly used for the adsorption of dyes and heavy metals [15]. Chitosan-based hydrogels have emerged as a superior alternative to traditional adsorbents for removing various dyes, given their non-toxicity, ease of separation, recyclability, and biodegradability [16]. In recent years, there have been noteworthy advancements in research focusing on the modification of chitosan hydrogels for organic pollutant degradation. Compared to linear chitosan, chitosan hydrogel displays larger pore sizes and a more expansive specific surface area, achieving an adsorption capacity of up to 187.5 mg·g^−1^. Kang et al. [17] developed a montmorillonite nanoplate/chitosan hydrogel to remove methylene blue from water, showcasing a peak adsorption capacity of 530 mg·g^−1^.

This study presents the synthesis of polyvinyl alcohol/carboxylated chitosan (PVA/CCTS) hydrogel using a free radical polymerization technique as an effective adsorbent for the removal of RhB from aqueous solutions [18]. The charge characteristics of CCTS notably improve the adsorption efficacy of the material, while the porous nature of the PVA/CCTS composite structure augments the specific surface area, offering a plethora of active sites for pollutant capture. This research seeks to do the following: (1) Develop a simplified method for preparing PVA/CCTS hydrogels. (2) Characterize the materials using a series of techniques (including Fourier-transform infrared spectroscopy (FTIR), scanning electron microscopy (SEM), X-ray photoelectron spectroscopy (XPS), and Brunauer–Emmet–Teller (BET) specific surface area analysis). (3) Investigate the effects of various parameters on the hydrogel’s adsorption performance under different environmental conditions, and conduct an in-depth analysis of the adsorption behavior and its underlying mechanisms through adsorption isotherm modeling, kinetic studies, and thermodynamic evaluations. (4) Conduct a detailed investigation of the adsorption mechanism of RhB on the hydrogel through theoretical calculations (including density functional theory (DFT) and molecular dynamics (MD) simulations).

## 2. Results and Discussion

### 2.1. Characterization of PVA/CCTS Hydrogel

**Figure 1:**
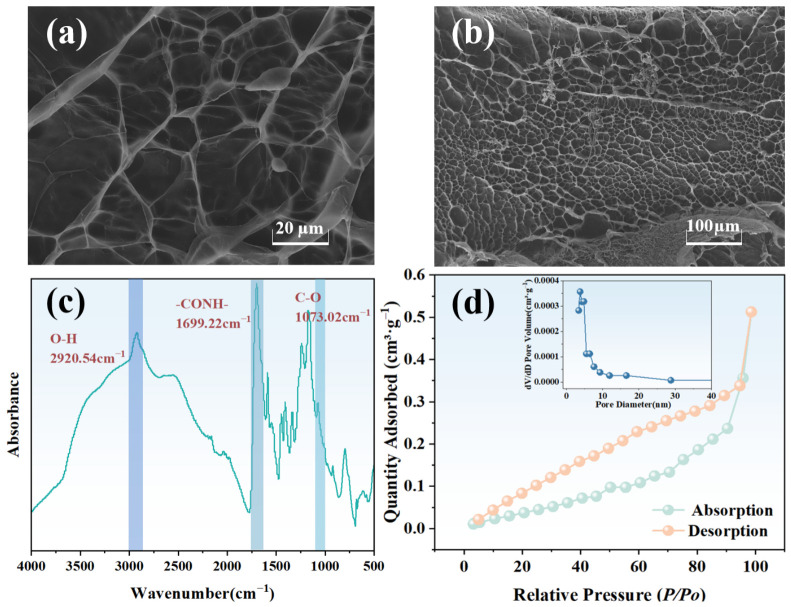
(**a**) SEM image of the hydrogel (×1000); (**b**) SEM image of the hydrogel (×200); (**c**) FTIR spectrum of the hydrogel; (**d**) The adsorption–desorption isotherm and the corresponding pore size distribution.

As depicted in Figure 1a,b, PVA/CCTS hydrogels exhibit a distinct and interconnected porous network structure. Their surfaces are smooth, revealing numerous circular or elliptical pores at various magnifications. These pores are uniformly distributed with thin pore walls. The FTIR spectrum in Figure 1c distinctly features a spectral band at 2920.54 cm^−1^, indicative of the presence of O-H groups. The spectral band at 1073.02 cm^−1^ is directly related to the stretching vibration of C-O bonds in hydrogel molecules [19]. The spectral band appearing at 1699.22 cm^−1^ in PVA/CCTS hydrogel indicates the presence of -CO-NH- groups [20]. The common peak band at 1056 cm^−1^ disappears in the hydrogel spectrum, indicating that during the free radical polymerization process, acrylic monomers have been successfully crosslinked onto the polyvinyl alcohol backbone, forming a new and stable chemical structure.

The pore size distribution in Figure 1d was derived from the N_2_ desorption branch using the Barrett–Joyner–Halenda model based on the Kelvin equation, which is appropriate for the mesoporous range identified by the Type-IV isotherm with H3-type hysteresis. Table 1 shows that the specific surface area of the hydrogel is 0.56 m^2^·g^−1^, which is relatively small. This material exhibits a mesoporous-dominated porous structure with an average pore diameter of 7.31 nm and a low microporous content.

**Figure 2:**
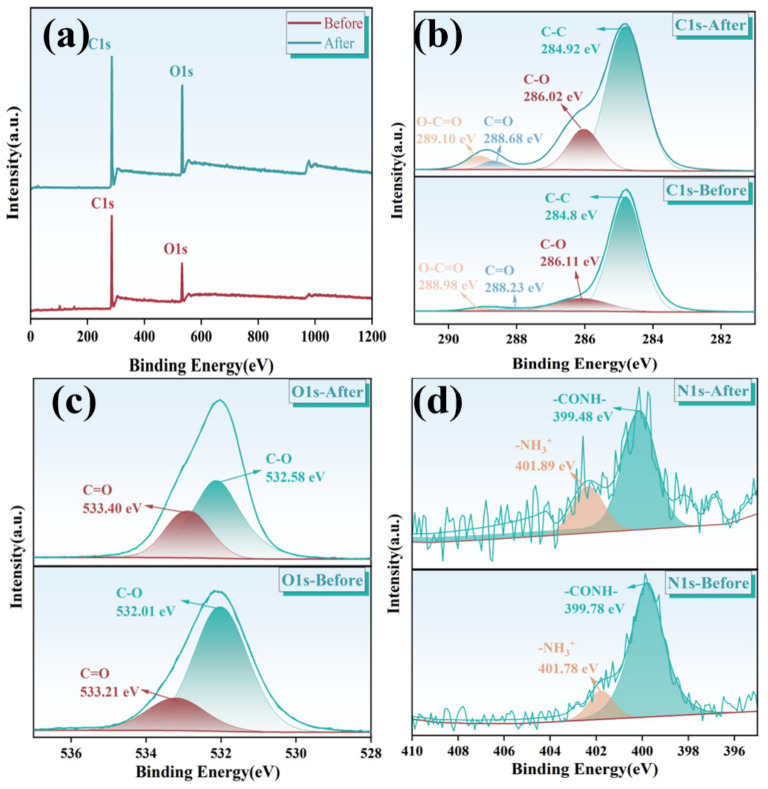
XPS analysis of PVA/CCTS hydrogel before (**lower panel**) and after (**upper panel**) RhB adsorption: (**a**) full survey spectra; (**b**) C 1 s; (**c**) O 1 s; (**d**) N 1 s. (In (**b**–**d**), the noisy trace is the raw spectrum, the solid line is the cumulative fit, and the shaded peaks are the deconvoluted components).

The full XPS spectrum, depicted in Figure 2a, reveals the characteristic peaks of C and O elements. Subsequent to RhB adsorption, alterations in these elements are perceptible in the spectrum. The PVA/CCTS hydrogel incorporates amide groups, while RhB comprises amino groups, allowing for the detection of element N both pre-adsorption and post-adsorption. In Figure 2b, peaks at binding energies of 284.8 eV, 286.11 eV, 288.23 eV, and 288.98 eV represent C-C, C-O, C=O, and O-C=O bond energies, respectively. It can be seen that after adsorption, there are no new peaks of C and O elements before and after adsorption, which proves that the adsorption of RhB by hydrogel depends on hydrogen bonding and electrostatic interaction. The binding energy of C-C bond increases slightly (+0.11 eV), the binding energy of the C-O bond experienced a significant increase, while the binding energy of the C=O bond exhibited only a slight uptick. This can be attributed to the enhanced electron density of the C-O bond, potentially facilitating binding to the amino or hydroxyl groups of RhB via hydrogen bonding or electrostatic interaction. Furthermore, the peaks at 532.01 eV and 533.21 eV in Figure 2c are indicative of C-O and C=O bond energies. Lastly, the peaks at 399.78 eV, 401.78 eV, and 402.88 eV in Figure 2d correspond to the bond energies of -CONH-, -NH_3_^+^ and -NO_2_, respectively. The -CONH- bond binding energy is reduced (−0.3 eV), which reflects the increase of the electron cloud density of the amide nitrogen, and the surface charge may be balanced by hydrogen bond or charge compensation [21]. The weak increase of -NH_3_^+^ bond (+0.11 eV) further confirms the electrostatic contribution of protonated amino groups [22].

### 2.2. Effect of Hydrogel Dosage

**Figure 3:**
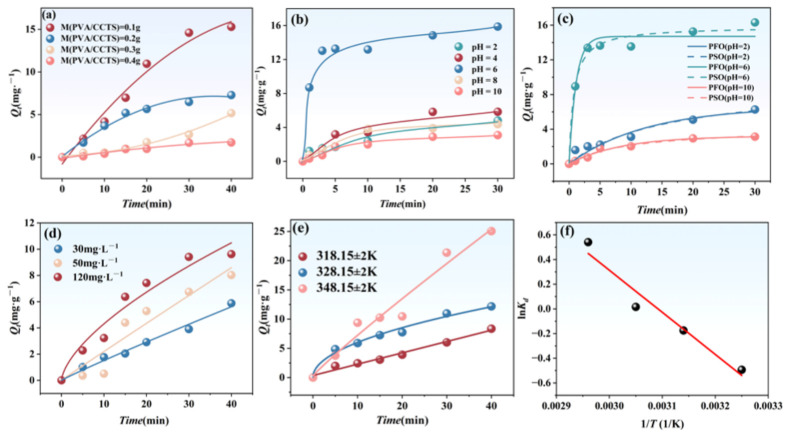
(**a**) The effect of adsorbent dosage on the *Q_t_* of RhB. Experimental conditions: *M* (PVA/CCTS) =0.1 g, 0.2 g, 0.3 g, 0.4 g. *t* = 0~40 min, *T* = 298.15 K ± 2 K, *C*_0_ = 65 mg·L^−1^. (**b**) The effect of pH on the *Q_t_* of RhB. Experimental conditions: pH = 2, 4, 6, 8, 10. *t* = 0~30 min, *T* = 298.15 K ± 2 K. (**c**) Kinetic fitting curve. Experimental conditions: pH = 2, 6, 10. *t* = 0~30 min, *T* = 298.15 ± 2 K. (**d**) The effect of concentration on the *Q_t_* of RhB. Experimental conditions: *C*_0_ = 30 mg·g^−1^, 50 mg·g^−1^, 120 mg·g^−1^, *t* = 0~40 min, *T* = 298.15 ± 2 K. (**e**) The effect of temperature on the *Q_t_* of RhB. *T* = 318.15 ± 2 K, 328.15 ± 2 K, 348.15 ± 2 K. *t* = 0~40 min. *C*_0_ = 65 mg·L^−1^. (**f**) Thermodynamic fitting.

Figure 3a demonstrates the impact of PVA/CCTS hydrogel dosage on the adsorption capacity for RhB. As the dosage of PVA/CCTS hydrogel increased, there was a corresponding gradual decrease in RhB’s adsorption rate. At the lowest dosage of 0.1 g, the hydrogel displayed superior adsorption performance, with an equilibrium adsorption capacity reaching 15.88 mg·g^−1^. However, at a dosage of 0.4 g, this capacity significantly diminished to 1.72 mg·g^−1^. This reduction in adsorption capacity can be attributed to two main factors. Firstly, excessive hydrogel can cause agglomeration of adsorbent particles, thereby reducing the effective adsorption surface area and consequently decreasing adsorption capacity and removal efficiency [23]. This observation aligns with previous studies on PVA-based hydrogels where similar effects of diffusion and saturation were noted with increasing dosage. Secondly, the increased quantity of adsorbent resulted in quicker adsorption saturation. Moreover, larger quantities of hydrogel elevated system viscosity, further decelerating the movement rate of RhB molecules in solution. Thus, employing a smaller mass of PVA/CCTS hydrogel not only enhances adsorption efficiency but also reduces treatment costs, offering practical advantages. For subsequent experiments, a dosage of 0.1 g was employed to ensure optimal adsorption performance.

### 2.3. Effect of pH and Adsorption Kinetics Analysis

pH significantly influences the molecular morphology of RhB and the surface charge properties of the hydrogel [24]. As shown in Figure 3b, the adsorption capacity exhibits a trend of first increasing and then decreasing with rising pH. When pH gradually increased from 2 to 6, the equilibrium adsorption capacity of PVA/CCTS hydrogel for RhB significantly increased from 4.82 mg·g^−1^ to 15.88 mg·g^−1^. However, as pH further increased, the equilibrium adsorption capacity exhibited a decreasing trend, ultimately dropping to 3.10 mg·g^−1^ at pH 10. This may be related to the ionization characteristics of the -COOH groups on the hydrogel molecular chains. In acidic environments, as pH rises, the dissociation of -COOH groups in the hydrogel is limited due to decreased H^+^ ion concentration [25]. Concurrently, the speciation of RhB in solution is governed by its acid dissociation constant (p*K*_a_ = 3.22) [26]: the protonated cationic form RhB^+^ predominates at pH < p*K*_a_, while the zwitterionic form RhB^±^ becomes dominant at pH > p*K*_a_, favoring electrostatic attraction toward the negatively charged hydrogel surface. Above pH 5, deprotonation of the carboxyl groups (p*K*_a_ of acrylic acid ≈ 4.25) further promotes hydrogel swelling and increases the accessibility of adsorption sites. However, under alkaline conditions, the hydrogel structure gradually dissociates and swells, making it more susceptible to damage and loss of adsorption capacity. Optimal adsorption occurs under neutral conditions, likely due to the best electrostatic matching between the adsorbent’s surface charge state and the ionic form of RhB [27]. Based on a comprehensive analysis of adsorption capacity, kinetic behavior, and mechanism, pH = 6 was determined as the optimal operating condition for subsequent experiments.

Kinetic fitting was performed at pH 2, 6, and 10 to evaluate the applicability of the pseudo-first-order (PFO) and pseudo-second-order (PSO) models (Table 2). At the optimal pH of 6, the PSO model provided a substantially better fit (*R*^2^ = 0.997 vs. 0.977), and its predicted equilibrium capacity (*Q*_e,cal_ = 16.6 mg·g^−1^) closely matched the experimental value (15.9 mg·g^−1^), whereas the PFO prediction (3.21 mg·g^−1^) deviated by ~80%. Although the PFO model showed marginally higher *R*^2^ values at pH 2 and 10, the predicted *Q*_e,cal_ at these conditions also diverged considerably from the experimental data (e.g., 14.7 vs. 3.10 mg·g^−1^ at pH 10), indicating limited physical validity for either model under extreme pH. Overall, the PSO model best fitted the adsorption kinetics under optimum conditions (pH = 6), indicating that RhB uptake involved complex interaction mechanisms including electrostatic attraction, hydrogen bonding and van der Waals forces rather than simple physisorption.

### 2.4. Effect of RhB Concentration and Isotherm Adsorption Analysis

The adsorption capacity (*Q_t_*) of RhB over time at different initial concentrations (30, 50, and 120 mg·L^−1^) is shown in Figure 3d. The figure indicates that adsorption capacity increases significantly with higher initial concentrations: at 30 mg·L^−1^, adsorption reached 5.88 mg·g^−1^ after 40 min, whereas at 120 mg·L^−1^, adsorption reached 9.62 mg·g^−1^ under the same time condition. This demonstrates that higher initial concentrations provide greater mass transfer driving forces, facilitating full utilization of adsorption sites. Overall, the initial concentration exerts a significant influence on adsorption capacity, consistent with the concentration effect characteristic typical of adsorption processes.

**Figure 4:**
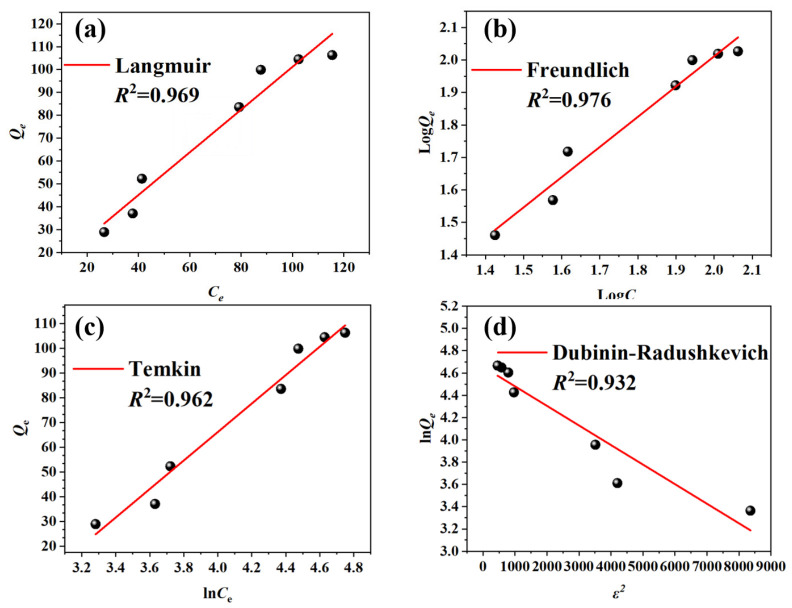
(**a**) Langmuir isotherm model fitting for the adsorption of RhB; (**b**) Freundlich isotherm model fitting for the adsorption of RhB; (**c**) Temkin isotherm model fitting for the adsorption of RhB; (**d**) Dubinin-Radushkevich (D-R) isotherm model fitting for the adsorption of RhB.

Adsorption isotherms characterize the types of interactions between adsorbates and adsorbent surfaces [28]. The adsorption mechanism was examined using four different models: Langmuir, Freundlich, Temkin, and Dubinin-Radushkevich (D-R). Upon integrating the data from Figure 4 with the findings presented in Table 3, it becomes evident that the Freundlich model, with an *R*^2^ value of 0.976, exhibits a superior fit compared to the Langmuir model, which has an *R*^2^ of 0.969. This suggests that the Freundlich model is better suited to describing the subtle differences in adsorption processes on heterogeneous gel surfaces, indicating that gel surfaces are likely to be heterogeneous [29]. Conversely, the D-R model exhibited the poorest fit, implying that the adsorption mechanism of RhB facilitated by PVA/CCTS hydrogel is not solely attributed to pore filling.

### 2.5. Effect of Temperature and Adsorption Thermodynamics Analysis

To further investigate the adsorption characteristics and evaluate the impact of temperature on them, we conducted adsorption experiments at temperatures of 318.15 ± 2 K, 328.15 ± 2 K, and 348.15 ± 2 K, with initial conditions set as pH = 6, *C*_0_ = 65 mg·L^−1^, and *M* = 0.1 g. As can be seen from Figure 3e, as the temperature increases, the adsorption capacity *Q_e_* gradually increases, reaching a maximum equilibrium adsorption capacity of 25.04 mg·g^−1^ at 348.15 ± 2 K, indicating that an increase in temperature is beneficial for improving adsorption efficiency.

The energy change and directionality during the adsorption process can be analyzed through thermodynamic parameters. As shown in Table 4, the Gibbs free energy changes (Δ*G*) at 308.15 ± 2 K, 318.15 ± 2 K, 328.15 ± 2 K, and 338.15 ± 2 K are 1.36 kJ·mol^−1^, 0.48 kJ·mol^−1^, −0.40 kJ·mol^−1^, and −1.28 kJ·mol^−1^, respectively. This indicates that the adsorption transitions from non-spontaneous to spontaneous around 324 K, in good agreement with the experimentally observed enhancement of adsorption capacity at elevated temperature. The enthalpy change (Δ*H*) is 28.38 ± 4.40 kJ·mol^−1^ > 0, indicating that the adsorption process is endothermic, meaning that an increase in temperature favors the adsorption of RhB [30]. The main mechanisms driving the adsorption are hydrophobic interactions and entropy increase, while an increase in temperature provides energy for the endothermic reaction. In addition, the entropy change (Δ*S*) is 87.73 ± 13.65 J·K^−1^·mol^−1^ > 0, indicating that the disorder of the system increases during the adsorption process, which also reflects the promoting effect of hydrophobic interactions in the endothermic process. In summary, an appropriate temperature increase is crucial for adsorption experiments.

### 2.6. Fixed-Bed Adsorption Experiments and Determination of Heat of Combustion

**Figure 5:**
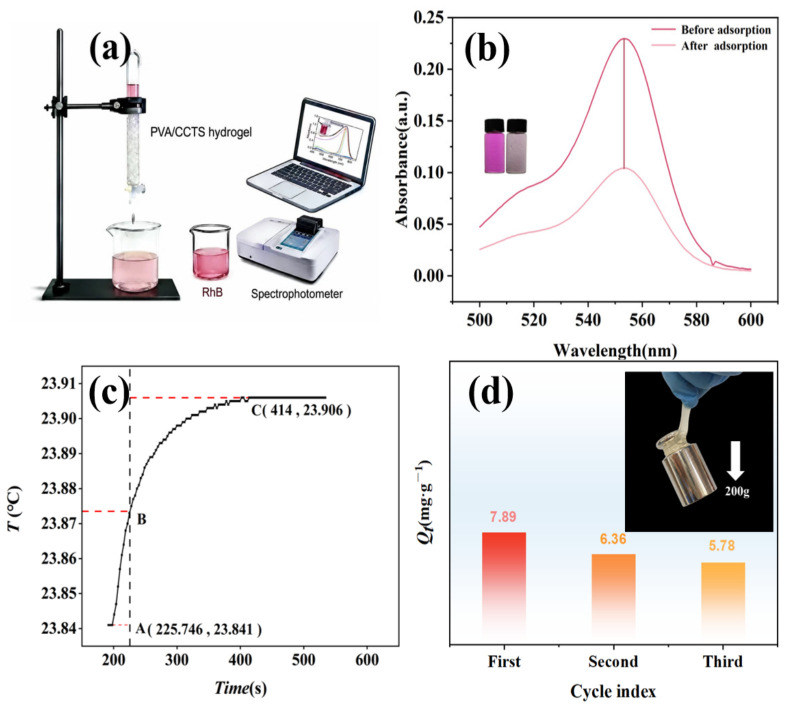
(**a**) Schematic diagram of the adsorption experiment of RhB solution by PVA/CCTS hydrogel; (**b**) UV-Vis absorption spectra of RhB solution before and after adsorption; (**c**) determine the temperature variation curve of the system during the combustion heat measurement process; (**d**) variation of adsorption capacity of PVA/CCTS hydrogel during three adsorption–desorption cycles; inset shows a load-bearing test.

The stability of adsorbents is a core indicator for evaluating their practical application. Fixed-bed column adsorption experiments were conducted to simulate the remediation process of wastewater contaminated with RhB. The adsorption of RhB solution by the PVA/CCTS hydrogel is illustrated in Figure 5a, where the solution color transitions from dark to light, visually demonstrating the hydrogel’s adsorption capacity for RhB. Figure 5b displays the absorbance change of the RhB solution before and after adsorption, with a reduction of 54%, demonstrating high application potential. As shown in Figure 5c, for spent hydrogels that can no longer be regenerated, bomb calorimetry measurements revealed a heating value of 9.39 kJ·g^−1^, confirming the feasibility of energy recovery as an alternative biomass fuel and effectively avoiding the potential environmental risks associated with direct landfill disposal [31].

### 2.7. Circulating Experiments and Material Comparison

From an economic standpoint, the recyclability and stability of an adsorbent are key factors in evaluating its practical utility. To gauge the chemical stability of PVA/CCTS hydrogel materials, a series of cycling experiments were performed on the adsorbent. Firstly, 0.1 g of PVA/CCTS hydrogel was adsorbed with 50 mg·L^−1^ RhB solution under conditions of pH 6 and temperature 298.15 ± 2 K, and its adsorption capacity was tested. After each adsorption experiment, the adsorbed hydrogel was immersed in a 0.1 mol·L^−1^ HCl solution for 6 h for desorption testing. After desorption, it was washed several times with ultrapure water until neutral, then reused under identical conditions. The experimental results are shown in Figure 5d; after three adsorption–desorption cycles, the adsorption capacities of PVA/CCTS for RhB were 7.89 mg·g^−1^, 6.36 mg·g^−1^, and 5.78 mg·g^−1^, respectively. This shows that PVA/CCTS has good mechanical properties and can be used repeatedly. Additionally, a comparison was conducted with previously reported adsorbent materials in Table 5, demonstrating that the PVA/CCTS hydrogel outperforms other materials in terms of RhB adsorption capacity. In summary, the PVA/CCTS hydrogel demonstrates promising scalability and industrial feasibility due to its exceptional recyclability and superior adsorption performance.

### 2.8. Analysis of Adsorption Mechanism

**Figure 6:**
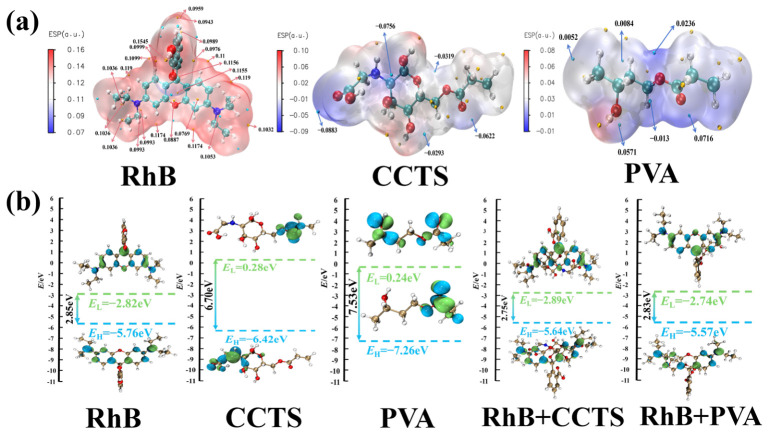
The electronic and interactional characteristics of the adsorption systems: (**a**) molecular electrostatic potential (ESP) analysis of the hydrogel surface; (**b**) Homo-Lumo orbital distribution and bandgap of CCTS and PVA monomers versus their Homo-Lumo orbital distribution and bandgap after RhB adsorption (white: H, cyan: C, blue: N, red: O).

Given the high similarity between the chemical structures of monomers and polymer repeating units, Two representative monomers of CCTS and PVA can be used in computational simulations to directly investigate their interaction mechanisms with RhB in place of the entire hydrogel. Figure 6a reveals that RhB exhibits overall positive charge, with its surface electrostatic potential predominantly red. The -OH and H groups on its ring represent the most positively charged regions. The CCTS surface shows overall negative charge, with -COOH being the most negatively charged site (−0.09 a.u.). The most negatively charged region on the PVA surface is −0.01 a.u. These charge distribution characteristics indicate that RhB adsorption onto hydrogels is primarily driven by electrostatic interactions between the -OH and H groups on the RhB ring and the -COOH groups in the hydrogel. Figure 6b shows that the HOMO-LUMO energy levels and orbital distributions of each system undergo significant changes after adsorption, directly confirming the occurrence of pronounced electron transfer between RhB and the substrate.

**Figure 7:**
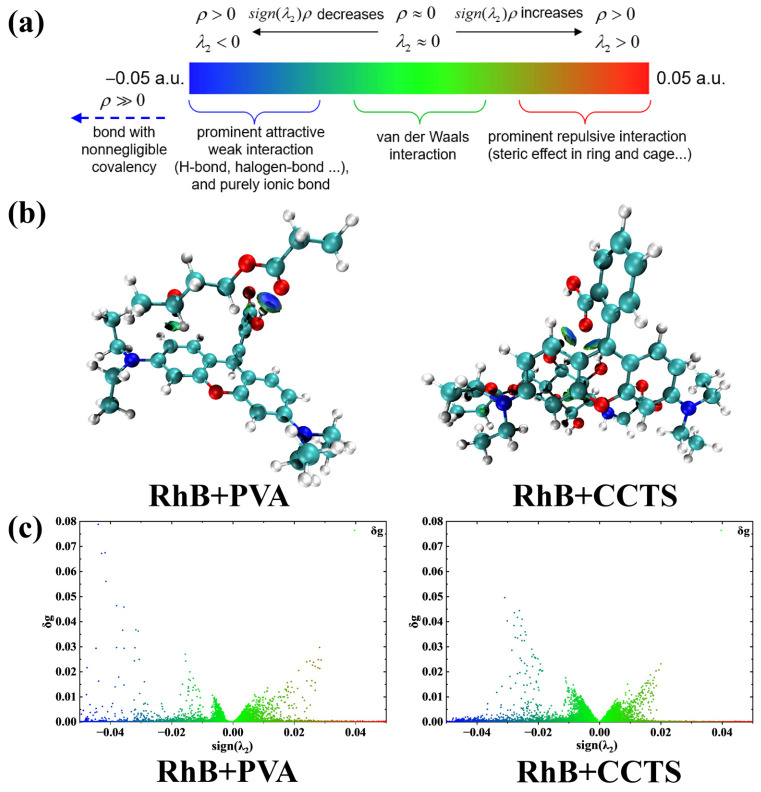
(**a**) Color legend of the IGMH analysis; (**b**) visualization of weak interaction regions for different adsorption configurations (white: H, cyan: C, blue: N, red: O); and (**c**) scatter plots of weak interactions between each substrate and RhB.

The IGMH analysis in Figure 7 visualizes the weak interactions during adsorption, where both PVA and CCTS primarily interact with RhB via van der Waals forces while simultaneously forming hydrogen bonds with RhB. These two interactions act synergistically to realize efficient RhB adsorption. Combined with the adsorption energy data in Table 6, it is further confirmed that the hydrogel’s adsorption of RhB is dominated by physical adsorption at the molecular level. In summary, the adsorption of RhB onto the hydrogel arises from the combined effects of electrostatic interactions, electron transfer and weak interactions (van der Waals forces and hydrogen bonding). PVA and CCTS jointly act as the functional units for RhB adsorption, and the synergy of van der Waals forces and hydrogen bonding underpins the high adsorption performance of the hydrogel for RhB.

**Figure 8:**
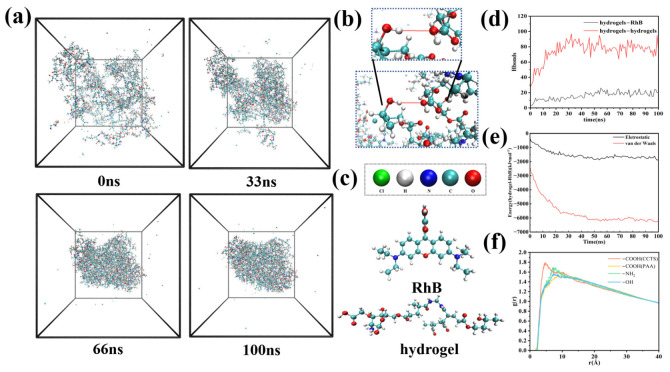
(**a**) Schematic of the simulated adsorption system; (**b**) snapshot of hydrogen bonding crosslinks between hydrogel chains; (**c**) molecular structures of RhB and hydrogels; (**d**) number of hydrogen bonds between hydrogels and between hydrogels and RhB; (**e**) force decomposition diagram of RhB adsorption by hydrogels; (**f**) radial distribution of RhB and various functional groups of hydrogels.

To elucidate the adsorption mechanism of RhB onto the hydrogel, 100 ns molecular dynamics (MD) simulations were performed. The simulation results showed that significant and stable adsorption of RhB occurred on the hydrogel surface. As shown in Figure 8d, hydrogen bond analysis indicated that hydrogel chains preferentially crosslinked via intermolecular hydrogen bonds during the formation of the three-dimensional network structure. Meanwhile, stable hydrogen bond interactions formed between hydrogel chains and RhB molecules, providing a key driving force for RhB adsorption. Energy decomposition results in Figure 8e further revealed that the adsorption process was dominated by van der Waals forces, with electrostatic forces acting as a supplement. In addition, radial distribution function (RDF) analysis (Figure 8f) confirmed that RhB preferentially interacted with carboxyl (-COOH) and amino (-NH_2_) active sites on the hydrogel surface, clarifying the adsorption sites and core mechanism at the molecular level.

In summary, molecular dynamics simulations clearly revealed the adsorption mechanism of RhB onto the hydrogel: the hydrogel forms a stable three-dimensional network through intermolecular hydrogen bonds and achieves efficient RhB adsorption via hydrogen bonds, van der Waals forces, and electrostatic interactions, among which van der Waals forces play a dominant role. Meanwhile, RhB mainly interacts with carboxyl and amino active sites on the hydrogel surface. These results are consistent with DFT calculations and jointly elucidate the adsorption mechanism of RhB onto the hydrogel.

## 3. Materials and Methods

### 3.1. Chemical Reagents

RhB (C_28_H_31_ClN_2_O_3_, purity ≥ 95.83%), chitosan (CS: molecular weight~560,000 g·mol^−1^, deacetylated 80.0–95.0%, viscosity 50–800 mPa·s), polyvinyl alcohol (PVA: molecular weight~200,000 g·mol^−1^, viscosity 54.0–66.0 mPa·s, alcoholysis degree ≥ 98.0%), acrylic acid (C_3_H_4_O_2_, AA: purity ≥ 98%), ammonium persulfate ((NH_4_)_2_S_2_O_8_, APS), N,N′-methylenebisacrylamide (MBA, C_7_H_10_N_2_O_2_), sodium carbonate (Na_2_CO_3_), 1-chloroacetic acid (CH_2_ClCOOH, purity ≥ 98%), isopropanol (C_3_H_8_O, purity ≥ 99.7%), and sodium hydroxide (NaOH) were purchased from Sinopharm Chemical Reagents Co., Ltd. (Shanghai, China). All deionized water required for the experiment was prepared in ultrapure water equipment (ZC1-A10Y, Changsha Zhichuang, Changsha, China).

### 3.2. Synthesis Steps

#### 3.2.1. Preparation of Carboxylated Chitosan (CCTS)

The preparation process was conducted as follows: (1) First, 0.5 g of chitosan (CS) was combined with 8.5 mL of isopropanol in a beaker and allowed to swell for 1 h under normal temperature and pressure. (2) Subsequently, 2.5 g of NaOH was added, followed by ultrasonic oscillation for 5 min. The mixture was then subjected to an alkaline reaction for 4 h. (3) After this, 1.5 g of 1-chloroacetic acid was introduced and the mixture was ultrasonically oscillated for an additional 5 min. The carboxylation process proceeded for 2 h, maintained at a reaction temperature of 333.15 ± 2 K. (4) Finally, the resultant solution was placed in an oven set at 323.15 ± 2 K for 2 h to dry, yielding a white carboxylated chitosan (CCTS).

#### 3.2.2. Preparation of PVA/CCTS Hydrogel

**Figure 9:**
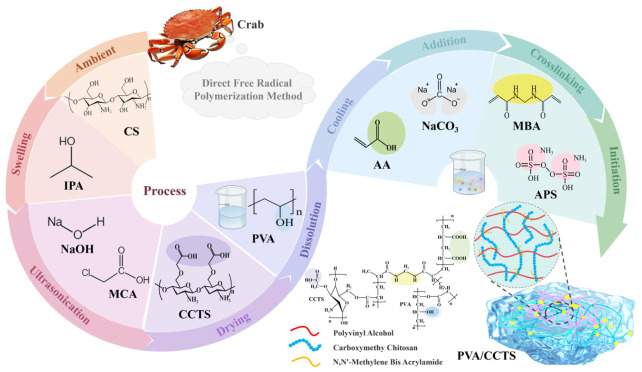
The simple preparation process of PVA/CCTS hydrogel and adsorption experiment.

PVA/CCTS hydrogels were synthesized using a straightforward free radical polymerization method. The specific preparation steps were as follows: (1) 0.5 g of PVA was dissolved in 7.0 mL of deionized water in a beaker to produce a clear solution. (2) To this solution, 0.23 g of CCTS was added, and after a 5 min cooling period, 2 mL of AA and 0.23 g of Na_2_CO_3_ were introduced. (3) Subsequently, 0.015 g of the MBA crosslinker and 0.015 g of the APS initiator were incorporated and thoroughly mixed. (4) The mixture was then subjected to a crosslinking reaction at 333.15 ± 2 K for 2 h, during which the CCTS interpenetrated with the PVA, resulting in the formation of the PVA/CCTS hydrogel. The simple preparation process of PVA/CCTS hydrogel and the adsorption experiment procedure are illustrated in Figure 9.

### 3.3. The Computational and Experimental Procedures

#### 3.3.1. Adsorption Kinetics

Adsorption kinetics describes the rate of adsorbate uptake by adsorbents. The pseudo-first-order, pseudo-second-order are as follows [36,37,38,39]:

Pseudo-first-order kinetic:

(1) $$Q_{t}=Q_{e}\left(1-exp\left(-k_{1}t\right)\right)$$

Pseudo-second-order kinetic:

(2) $$t/Q_{t}=t/Q_{e}+1/k_{2}Q_{e}^{2}$$

where *Q_e_* and *Q_t_* are the equilibrium adsorption amount and the adsorption amount of RhB by the hydrogel at time *t*, respectively, mg·g^−1^. *k*_1_ is the rate constant of pseudo-first-order reaction, min^−1^. *k*_2_ is the rate constant of pseudo-second-order reaction, g·mg^−1^·min^−1^.

#### 3.3.2. Isotherm Adsorption

Adsorption isotherms were used to analyze adsorption behavior via linear fitting of three models: Langmuir [40], Freundlich [41], Temkin, and Dubinin-Radushkevich [42], to determine monolayer/multilayer adsorption and homogeneous/heterogeneous surface interactions.

Langmuir model:

(3) $$C_{e}/Q_{e}=1/bQ_{m}+C_{e}/Q_{m}$$

Freundlich model:

(4) $$Q_{e}=K_{f}C_{e}^{\frac{1}{n}}$$

Temkin model:

(5) $$Q_{e}=\frac{RT}{b_{T}}\ln(\alpha_{T}C_{e})$$

Dubinin-Radushkevich model:

(6) $$Q_{e}=Q_{m}\varepsilon^{2}exp\left(-\beta \right)$$

(7) $$\varepsilon =RT\ln(1+1/C_{e})$$

(8) $$E=2\beta^{-\frac{1}{2}}$$

*C_e_* denotes the concentration at adsorption equilibrium, mg·L^−1^. *b* is the adsorption coefficient related to adsorption performance, L∙mg^−1^. *K_f_* is the Freundlich affinity coefficient, representing the binding strength of the adsorbent and adsorbate. *n* is the Freundlich constant. *α_T_* is the Temkin equilibrium binding constant, L·g^−1^. *b_T_* is the Temkin constant related to heat of adsorption, kJ·moL^−1^. *β* is a constant related to the average adsorption free energy per mole of RhB, mol^2^·J^−2^. *ɛ* is the Polanyi potential. *R* is the gas constant, J·mol^−1^·K^−1^. *T* is the temperature, K. *E* is the average free energy, kJ·mol^−1^.

#### 3.3.3. Adsorption Thermodynamics

Thermodynamic parameters can accurately reflect the spontaneous characteristics of reactions, while studying the dynamic change of adsorption capacity with temperature is an important dimension to deeply analyze the adsorption ability. In the adsorption process, the thermodynamic equilibrium constant (*K_d_*), Gibbs free energy change (Δ*G*), enthalpy change (Δ*H*), and entropy change (Δ*S*) of the adsorption reaction can be calculated as follows:

(9) $$∆G=-RT\ln K_{d}$$

(10) $$K_{d}=Q_{e}/C_{e}$$

(11) ∆G=∆H−T∆S

where *K_d_* is the partition coefficient. Δ*G* is the Gibbs free energy change of adsorption, kJ∙mol^−1^. Δ*H* is the enthalpy change of adsorption, kJ∙mol^−1^. Δ*S* is the change in adsorption entropy, J·K^−1^·mol^−1^.

#### 3.3.4. Adsorption Experiment

A certain mass of PVA/CCTS hydrogels were placed in deionized water, respectively. After reaching equilibrium swelling, they were added to 200 mL of RhB solution with concentrations of (30, 50, 120 mg·L^−1^), and the pH value was adjusted to achieve levels of (2, 4, 6, 8, 10). The mixture was shaken at a speed of 200~250 rpm at 298.15 ± 2 K (except for experiments studying temperature effects). Liquid samples were collected at fixed time intervals (1, 3, 5, 10, 20, 30, 40 min). The samples were filtered through a Millipore membrane filter (0.22 μm), and the RhB concentration was measured using an ultraviolet spectrophotometer (OPLE-1800 model, Shanghai Opu Instrument Co., Ltd., Shanghai, China). Then, 200 mL of RhB dye solution containing hydrogels at different concentrations was stirred at room temperature to test the adsorption capacity of the hydrogels. All experiments were conducted three times, and the average values were calculated.

The adsorption capacity was calculated according to Equations (12) and (13) [43]. (*Q_e_*, mg·L^−1^) and removal efficiency [15] (RR, %):

(12) $$Q_{e}=\left(C_{0}-C_{t}\right)\times V/M$$

(13) $$RR=(C_{e}-C_{t})/C_{t}\times 100\%$$

where *V* is the solution volume, L. *m* denotes the mass of dry hydrogel, g. *RR* signifies the removal efficiency; *C*_0_ and *C_t_* correspond to the initial concentration and concentration at a specific time point, mg·L^−1^.

A stock solution of RhB was prepared at a concentration of 50 mg·L^−1^, which was subsequently diluted to produce standard solutions with concentrations of 0.1, 0.2, 0.4, 0.8, 1.0, 1.2, 1.6, and 2.0 mg·L^−1^. The wavelength scanning was conducted within the range of 500–600 nm. The absorbance of the RhB solutions at a wavelength of 552 nm was then plotted against the concentration to generate the standard curve. This yielded the fitting equation: *Y* = 0.01195*X* + 0.00443 (*R*^2^ = 0.9999).

#### 3.3.5. Fixed-Bed Adsorption Experiments

To evaluate the removal effect of PVA/CCTS hydrogel on RhB in water bodies, a dynamic adsorption experiment was conducted using a fixed-bed column. A glass column with a length of 20 cm and a diameter of 20 mm was filled with 0.2 g of fully swollen hydrogel samples. A 30~40 mL solution of 50 mg·L^−1^ RhB was added to the column to simulate industrial wastewater. Dynamic adsorption was carried out at room temperature and a flow rate of 0.2 mL·s^−1^. Finally, the concentration of RhB in the solution before and after the cycle was detected using UV–visible spectrophotometry to evaluate the adsorption performance of the hydrogel.

#### 3.3.6. Cycle Experiment

Place 0.1 g dried PVA/CCTS hydrogel in deionized water, and after reaching swelling equilibrium, add an RhB solution with a concentration of 50 mg·L^−1^. Once adsorption equilibrium is achieved, remove the hydrogel and use 20 mL of eluent with concentrations ranging from 0.1 mol·L^−1^ to 0.3 mol·L^−1^ HCl solution for desorption experiments in a constant temperature oscillator.

#### 3.3.7. Determination of Heat of Combustion

The combustion heat of PVA/CCTS hydrogel was measured using an oxygen bomb calorimeter (XRY-1A, Shanghai Precision Instrument, Shanghai, China). A 0.05 g sample was pelleted, placed inside the bomb, and contacted with an iron-wire electrode. The bomb was filled with high-pressure oxygen, immersed in a calorimeter with a known volume of water, and equipped with a stirrer and thermometer. After temperature stabilization, the sample was ignited electrically. The water temperature rise during combustion was recorded until it peaked and declined. Combustion heat was calculated based on the calorimeter constant and the observed temperature change.

(14) $$Q_{v}=(C∆T-qm_{0})/mT$$

(15) $${∆}_{c}H_{m}^{\theta }=Q_{v}M$$

where *Q_V_* is the constant-volume combustion heat, J·g^−1^. *C* represents the heat capacity of the calorimeter, J·K^−1^. ∆*T* denotes the temperature change before and after combustion, K. *q* refers to the combustion heat of the ignition wire, J·g^−1^. *m*_0_ is the mass of the ignition wire; *m* indicates the mass of the substance under measurement, g. ∆$H_{m}^{\theta }$ represents the standard combustion heat of the substance, J·mol^−1^. *M* signifies the molar mass of the measured substance, g·mol^−1^.

### 3.4. Characterization

The material’s structure and chemical bonds were characterized by a Fourier transform infrared spectrometer (FTIR, Nexus 670, hermo Nicolet/Thermo Electron Corporation, Madison, WI, USA). The hydrogel’s surface morphology and microstructure were observed via a scanning electron microscope (SEM, JSM-7900F, JEOL Ltd., Akishima, Tokyo, Japan). The hydrogel’s surface chemical composition and valence states were determined by X-ray photoelectron spectroscopy (XPS, PHI 5000, Physical Electronics Inc., Chanhassen, MN, USA) using a 300 W monochromatic Al-Kα source. The Brunner–Emmett–Taylor (BET, Autosorb6300, Physical Electronics Inc., Chanhassen, MN, USA) analyzer was employed to determine the specific surface area, total pore volume, and pore size distribution of the material.

### 3.5. Theoretical Calculation

This study employs density functional theory (DFT) to investigate the adsorption behavior and interaction mechanism of RhB on PVA/CS and PVA/CCTS hydrogel. Given computational limitations, monomer fragment models were used instead of the full three-dimensional cross-linked hydrogel structure, which may ignore steric effects, solvation, polymer chain flexibility, and synergistic interactions of functional groups. However, this simplification is reasonable: full DFT calculations on the entire cross-linked network are computationally unaffordable, and using representative monomer segments as simplified models is a mature and common approach in adsorption-related research [44,45]. All structural optimizations and frequency calculations of the adsorption substrates were carried out using Gaussian 16 W software at the B3LYP/6-31G(d’) level; while single-point energy calculations were performed at the M062X/DEF2TZVP level [46,47,48,49,50,51].

To conduct an in-depth analysis of interaction characteristics, the study utilizes the wave function analysis software Multiwfn (Version 3.8.dev) to systematically analyze the molecular electrostatic potential (ESP) [52,53,54,55,56] based on the Independent Gradient Model (IGMH) with Hirshfeld partitioning and frontier molecular orbitals (FMOs). Additionally, the Visual Molecular Dynamics (VMD) software (version 1.9.3) is employed to visualize the electrostatic potential distribution, frontier molecular orbitals, and weak interactions during the PVA/CCTS adsorption of RhB [57]. The adsorption energy (*E_ad_*) between RhB and various adsorption substrates is calculated using Formula (16).

(16) $$E_{ad}=E_{ab}-E_{a}-E_{b}$$

where *E_ab_* is the total energy of the adsorption complex, and *E_a_* and *E_b_* represent the total energy of the adsorbent and adsorbate, respectively. The higher the absolute value of the binding energy, the more stable the adsorption system.

In MD simulations, the Gaussian 16 code was employed to optimize molecular structures using the B3LYP-D3 functional and the 6-31G(d) basis set. Subsequently, Multiwfn was utilized to fit the constrained electrostatic potential (RESP) charges. Water molecules employed the SPC water model [58]. Bond and non-bond parameters for other molecules were obtained using the sobtop software (Version 1.0.dev5) [59]. MD simulations were performed using the GROMACS software package version 2025.2 [60]. Molecules were described using the GAFF force field [61]. During simulation, 30 molecules and 30 hydrogel molecules were randomly added to an 8 × 8 × 8 nm^3^ simulation box. The box was then filled with water molecules, and the system charge was neutralized with Cl^−^ to construct the simulated system. Molecular dynamics simulations were performed using the same methodology. The initial contact problem was resolved using the steepest descent method to minimize the energy of each system. Short-time simulations of 500 ps were conducted under NVT and NPT ensemble distributions. Subsequently, positional constraints on the molecules were released, and a continuous 100 ns data-production run was performed using the NPT ensemble. Pressure was maintained at *p* = 1.0 bar, while temperature was controlled at 300 K via a velocity-scale thermostat with coupling constant *τ* = 0.1 ps. Non-bonded interactions were calculated with a cutoff of 1.2 nm, and long-range electrostatic interactions were computed using the particle-grid Ewald summation method. All hydrogen bonds were calculated using the LINCS algorithm [62]. The simulation time step was set to 2 fs, with the neighbor list updated every 10 steps. Periodic boundary conditions were applied in all three directions. Visualization was performed using VMD [57].

## 4. Conclusions

This study successfully synthesized a PVA/CCTS hydrogel, demonstrating superior RhB adsorption for environmentally friendly wastewater treatment applications. Under specific conditions—addition amount of 0.1 g, pH = 6, RhB concentration of 65 mg·L^−1^, and 298.15 ± 2 K—the PVA/CCTS hydrogel exhibited high-efficiency adsorption for RhB, achieving an adsorption capacity of 15.88 mg·g^−1^. Kinetic modeling revealed that the pseudo-second-order model provided the best fit under optimal conditions (*R*^2^ = 0.997), suggesting that the adsorption of RhB onto PVA/CCTS hydrogels involves a synergistic combination of electrostatic interactions, hydrogen bonding, and van der Waals forces. The Freundlich model fitting results (*R*^2^ = 0.976) indicate the surface heterogeneity of the hydrogel. Thermodynamic analysis confirms that the adsorption process is endothermic, (Δ*H* = 28.38 ± 4.40 kJ·mol^−1^, Δ*S* = 87.73 ± 13.65 J·K^−1^·mol^−1^). FTIR analysis revealed characteristic adsorption bands for O-H groups (2920.54 cm^−1^), C-O bonds (1073.02 cm^−1^), and -CO-NH- groups (1699.22 cm^−1^) within the PVA/CCTS hydrogel. XPS analysis further indicated shifts in binding energy for C, O, and N elements post-adsorption of RhB, providing insights into the reasons for these shifts and elucidating the adsorption mechanism between PVA/CCTS hydrogel and RhB, which primarily involves electrostatic adsorption and hydrogen bonding. DFT and MD analysis confirms that -COOH is the dominant RhB binding sites, with adsorption mainly driven by electrostatic, hydrogen-bonding, and van der Waals interactions, consistent with FTIR and XPS results and validating the theoretical simulations. The effective adsorption of RhB by PVA/CCTS hydrogel suggests not only a sustainable material for dye pollution control but also a promising avenue for complex wastewater treatment and industrial environmental remediation, given its potential for regeneration.

## Figures and Tables

**Table 1:** Pore structure parameters of the PVA/CCTS hydrogels.

Thermophysical Properties	BET Surface Area (m^2^·g^−1^)	Total Pore Volume (cm^3^·g^−1^)	DFT Cumulative Surface Area (m^2^·g^−1^)	DFT Mode Pore Diameter (nm)
CCTS/PVA	0.56 ± 0.04	0.003 ± 0.001	0.69 ± 0.06	7.3 ± 0.4

**Table 2:** Kinetic fitting results of RhB adsorption by PVA/CCTS hydrogel.

pH	Pseudo-First-Order			Pseudo-Second-Order			*Q_e_* (mg·g^−1^)
	*K*_1_ (min^−1^)	*R* ^2^	*Q_e_* (mg·g^−1^)	*K*_2_ (g·mg^−1^·min^−1^)	*R* ^2^	*Q_e_* (mg·g^−1^)	
2	0.081 ± 0.011	0.930	6.60 ± 0.15	0.0138 ± 0.0028	0.893	7.71 ± 0.25	4.82
6	0.118 ± 0.033	0.977	3.21 ± 0.50	0.0494 ± 0.0013	0.997	16.6 ± 0.22	15.9
10	0.889 ± 0.005	0.972	14.7 ± 0.07	0.0194 ± 0.0015	0.952	4.45 ± 0.11	3.10

**Table 3:** Isothermal adsorption fitting results.

Isotherm	Constants		
Langmuir	*Q_m_* (mg·g^−1^)	*K_L_* (L·mg^−1^)	*R* ^2^
	444.6 ± 224.7	0.00294 ± 0.00187	0.969
Freundlich	*K_F_* ((mg·g^−1^)·(L·mg^−1^)^(1/n)^)	*n*	*R* ^2^
	1.109 ± 0.503	1.000 ± 0.106	0.976
Temkin	*K_T_* (L·g^−1^)	*A* (mg·g^−1^)	*R* ^2^
	0.0569 ± 0.0073	58.54 ± 6.70	0.962
Dubinin- Radushkevich	*Q_m,D-R_* (mg·g^−1^)	*β* (mol^2^·J^−2^)	*R* ^2^
	108.90 ± 10.06	(2.08 ± 0.41) × 10^−4^	0.932

**Table 4:** Thermodynamics fitting results of RhB adsorption by PVA/CCTS hydrogel.

C_0_ (mg·L^−1^)	ΔH (kJ·mol^−1^)	ΔS (J·K^−1^·mol^−1^)	ΔG (kJ·mol^−1^)			
			308.15 ± 2 K	318.15 ± 2 K	328.15 ± 2 K	338.15 ± 2 K
65	28.38 ± 4.40	87.73 ± 13.65	1.36	0.48	−0.40	−1.28

**Table 5:** Comparison of the actual maximum adsorption capacity of RhB.

No.	Adsorbent	Maximum Adsorption Capacity, *Q_m_* (mg·g^−1^)	Preliminary RhB Concentration (mg·L^−1^)	Reference
1	Modified Clinoptilolite	20	2.81	[32]
2	biomass of cypress/false cypress (chamaecyparis lawsoniana) frui	1.180	10	[33]
3	Plant (Citrus Leaves)	0.28	8	[34]
4	Natural bamboo powder	7.64	20	[35]
5	polyvinyalcohol/carboxylated chitosan hydrogel	15.3	65	This study

**Table 6:** Adsorption energy values of RhB on different adsorption substrates.

Type	E_ab_/Hartree	E_a_/Hartree	E_b_/Hartree	E_ad_/kcal·mol^−1^
RhB + CCTS	−2507.116	−1420.168	−1086.948	−0.181
RhB + PVA	−1920.855	−1420.168	−500.681	−3.254

## Data Availability

Data are contained within the article.

## Abbreviations

The following abbreviations are used in this manuscript:

CCTS	Carboxymethyl chitosan
RhB	Rhodamine B
CS	Chitosan
PVA	Polyvinyl alcohol
FTIR	Fourier-transform infrared spectroscopy
SEM	Scanning electron microscopy
XPS	X-ray photoelectron spectroscopy
BET	Brunauer–Emmet–Teller
DFT	Density functional theory
MD	Molecular dynamics
PFO	Pseudo-first-order kinetic
PSO	Pseudo-second-order kinetic
D-R	Dubinin-Radushkevich
